# Formulation and Stability of Quercetin-Loaded Pickering Emulsions Using Chitosan/Gum Arabic Nanoparticles for Topical Skincare Applications

**DOI:** 10.3390/polym17131871

**Published:** 2025-07-04

**Authors:** Mathukorn Sainakham, Paemika Arunlakvilart, Napatwan Samran, Pattavet Vivattanaseth, Weeraya Preedalikit

**Affiliations:** 1 Department of Pharmaceutical Sciences, Faculty of Pharmacy, Chiang Mai University, Chiang Mai 50200, Thailand; mathukorn.s@cmu.ac.th (M.S.); paemika_a@cmu.ac.th (P.A.); napatwan_s@cmu.ac.th (N.S.); 2 Department of Pharmaceutical Sciences, School of Pharmaceutical Sciences, University of Phayao, Phayao 56000, Thailand; pattavet.vi@up.ac.th; 3 Department of Cosmetic Sciences, School of Pharmaceutical Sciences, University of Phayao, Phayao 56000, Thailand

**Keywords:** quercetin, pickering emulsions, chitosan, gum arabic, encapsulation, emulsion stability, biopolymer stabilizers, antioxidant stability

## Abstract

Natural polymer-based nanoparticles have emerged as promising stabilizers for Pickering emulsions, offering biocompatibility, environmental sustainability, and improved protection of active compounds. This study developed chitosan/gum arabic (CH/GA) nanoparticles as solid stabilizers for quercetin-loaded Pickering emulsions to enhance the stability and antioxidant bioactivity of quercetin (QE), a plant-derived flavonoid known for its potent radical-scavenging activity but limited by oxidative degradation. A systematic formulation strategy was employed to evaluate the effects of CH/GA concentration (0.5–2.0% *w*/*v*), oil type (olive, soybean, sunflower, and coconut), and oil volume fraction (ϕ = 0.5–0.7) on emulsion stability. The formulation containing 1.5% CH/GA and olive oil at ϕ = 0.6 exhibited optimal physical and interfacial stability. Quercetin (0.1% *w*/*w*) was incorporated into the optimized emulsions and characterized for long-term stability, particle size, droplet morphology, rheology, antioxidant activity (DPPH), cytocompatibility, and intracellular reactive oxygen species (ROS) protection using HaCaT keratinocytes. The olive oil-based formulation (D1-QE) exhibited greater viscosity retention and antioxidant stability than its soybean-based counterpart (E2-QE) under both room temperature (RT) and accelerated heating–cooling (H/C) storage conditions. Confocal microscopy confirmed the accumulation of CH/GA nanoparticles at the oil–water interface, forming a dense interfacial barrier and enhancing emulsion stability. HPLC analysis showed that D1-QE retained 92.8 ± 0.5% of QE at RT and 82.8 ± 1.5% under H/C conditions after 30 days. Antioxidant activity was largely preserved, with only 4.7 ± 1.7% and 14.9 ± 4.8% loss of DPPH radical scavenging activity at RT and H/C, respectively. Cytotoxicity testing in HaCaT keratinocytes confirmed that the emulsions were non-toxic at 1 mg/mL QE and effectively reduced H_2_O_2_-induced oxidative stress, decreasing intracellular ROS levels by 75.16%. These results highlight the potential of CH/GA-stabilized Pickering emulsions as a polymer-based delivery system for maintaining the stability and functional antioxidant activity of QE in bioactive formulations.

## 1. Introduction

Quercetin (QE) is a widely studied flavonoid known for its potent antioxidant, anti-inflammatory, and immunomodulatory properties, making it particularly attractive for pharmaceutical and cosmetic applications [1,2]. In topical skincare, QE has demonstrated the ability to neutralize reactive oxygen species and modulate inflammation, contributing to skin barrier protection and anti-aging benefits [3]. However, its incorporation into dermal formulations is hindered by poor aqueous solubility, limited skin permeability, and significant susceptibility to oxidative degradation, even under ambient oxygen exposure, which leads to the loss of biological activity and formation of degradation by-products [4].

To overcome these limitations, several formulation strategies have been explored, including glycosylation [5,6], nanocrystallization [7], and encapsulation systems. Glycosylation can improve water solubility and stability, although it may compromise antioxidant activity in certain cases [8]. In contrast, quercetin nanocrystals have demonstrated enhanced antioxidant effects in biological models, although they often require high-energy processing and may present challenges with long-term physical stability [9,10]. Among these options, Pickering emulsions have gained increasing attention for their suitability in topical delivery systems, especially when formulation simplicity, physical robustness, and biopolymer compatibility are desired. In particular, they offer the dual advantage of stabilizing QE and enhancing its dermal bioavailability [11,12]. Unlike conventional surfactant-based emulsions, Pickering systems are stabilized by solid particles, which minimize skin irritation and enable sustained release [13,14]. Compared to conventional carriers such as liposomes or micelles, Pickering emulsions are especially advantageous in topical applications due to their superior resistance to coalescence, ease of formulation with natural materials, and ability to form protective interfacial layers that prolong active retention on the skin.

Natural biopolymers, such as polysaccharides, are particularly well-suited as Pickering stabilizers for cosmetics, offering biodegradability, biocompatibility, and functional versatility. Chitosan, a cationic polysaccharide derived from chitin, is widely recognized for its film-forming, antimicrobial, and mucoadhesive properties [15]. Gum arabic, an anionic heteropolysaccharide primarily composed of arabinogalactan–protein complexes, is valued for its exceptional water solubility, strong emulsifying capacity, and film-forming ability [16]. It exhibits excellent stabilizing performance by promoting electrostatic and steric repulsions between particles, thereby preserving colloidal stability and preventing nanoparticle agglomeration [17]. Owing to these properties, gum arabic has been effectively employed as a stabilizer in the fabrication of diverse nanostructures, including metal nanoparticles, protein nanoparticles, and liposomes [16]. Moreover, Gum arabic coatings provide a protective interfacial layer that enhances nanoparticle biocompatibility and allows for controlled release, supporting their role as a non-toxic, biodegradable delivery matrix [18]. Gum arabic has also been combined with chitosan to form polyelectrolyte nanoparticles for quercetin delivery, achieving a high encapsulation efficiency (>90%) and particle size of 267–493 nm via ionic gelation [19]. The combination of chitosan and gum arabic leads to synergistic interfacial behavior, which is particularly useful in Pickering emulsions. Electrostatic complexation between oppositely charged polymers forms stable colloidal particles that can irreversibly adsorb at oil–water interfaces, thereby enhancing emulsion stability through physical barrier formation. These synergistic effects have been demonstrated in previous studies involving hydrophobic compounds such as curcumin, cannabidiol, and essential oils [20,21], underscoring the broad applicability and functionality of chitosan/gum arabic (CH/GA) nanoparticles in stabilized delivery systems.

Although quercetin-loaded Pickering emulsions have been developed using other stabilizing systems—such as cellulose nanocrystals [22]—the application of CH/GA nanoparticles as Pickering stabilizers for cosmetic formulations, particularly for the dermal delivery of sensitive flavonoids like QE, remains underexplored. While self-stabilizing particles and nanocrystals have shown promise in food and pharmaceutical applications, they often lack the formulation flexibility and skin compatibility of externally prepared CH/GA nanoparticles. A recent review by Wang et al. [22] outlined quercetin’s bioactivity and encapsulation approaches, including Pickering emulsions; however, applications involving biopolymer-based nanoparticles like CH/GA, for topical delivery remain limited.

To the best of our knowledge, this is the first study to apply CH/GA nanoparticles as solid-stabilizing agents in Pickering emulsions designed for the dermocosmetic delivery of QE. This strategy exploits the interfacial synergy of CH/GA particles to stabilize the emulsion, protect QE against degradation, and improve its functional retention in topical formulations. Beyond cosmetic use, the enhanced stability of these systems may facilitate the development of topical or dermatological formulations aimed at delivering antioxidant benefits.

Accordingly, the present work aims to formulate and characterize QE-loaded Pickering emulsions using CH/GA nanoparticles. By systematically varying the nanoparticle concentration, oil phase type, and oil volume fraction, this study identified stable emulsion systems and evaluated their long-term physicochemical stability, antioxidant activity (via DPPH and intracellular ROS assays), and cytocompatibility using HaCaT human keratinocytes. These results provide a practical framework for developing biopolymer-based emulsions that preserve QE’s bioactivity, with strong potential for clean-label cosmeceutical and dermatological applications.

## 2. Materials and Methods

### 2.1. Materials

Chitosan (food grade, degree of deacetylation 90–95%) and gum arabic (99.78% purity) were obtained from Union Science, Thailand. Olive, soybean, sunflower, and coconut oils were purchased from NSG, Thailand (Bangkok, Thailand). Quercetin, 2,2- diphenyl-1-picryhydrazyl (DPPH), ascorbic acid, Nile red, and Nile blue were purchased from Sigma Aldrich (St. Louis, MO, USA). All reagents and chemicals were of analytical grade, unless otherwise specified.

### 2.2. Preparation of Pickering Emulsions Base

**Figure 1:**
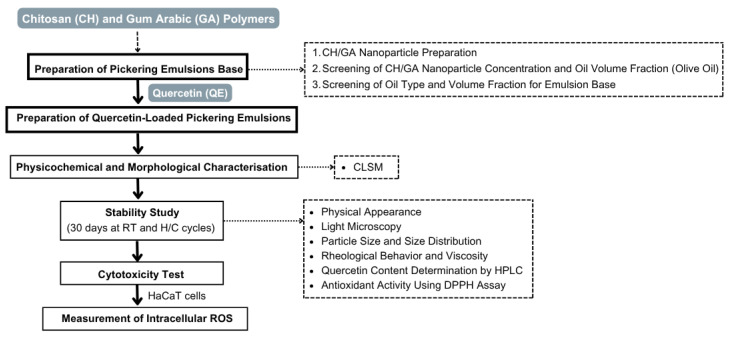
Schematic overview of the experimental workflow. Chitosan (CH) and gum arabic (GA) polymers were used to prepare CH/GA nanoparticles. Quercetin (QE) was incorporated to produce QE-loaded Pickering emulsions, followed by physicochemical and morphological characterization, including CLSM analysis, stability testing, cytotoxicity evaluation using HaCaT cells, and measurement of intracellular reactive oxygen species (ROS).

The preparation of the Pickering emulsion base was conducted in three main steps: (i) preparation of chitosan and gum arabic nanoparticles, (ii) screening of CH/GA nanoparticle concentration and oil volume fraction using olive oil, and (iii) screening of oil type and volume fraction for the Pickering emulsion base. These steps are summarized in the experimental workflow (Figure 1), with detailed methodologies provided in the following sections. 

#### 2.2.1. Chitosan and Gum Arabic Nanoparticle Preparation

CH/GA nanoparticles were prepared at a 1:1 ratio by first dissolving chitosan in 50 mL of 0.1 N acetic acid and gum arabic in 50 mL of deionized water (DI water). The gum arabic solution was then added gradually, dropwise, to the chitosan solution under continuous stirring at 800 rpm using a magnetic stirrer for 30 min at room temperature (25 ± 2 °C). The final suspension was used as the stabilizing phase of the emulsions.

#### 2.2.2. Screening of CH/GA Nanoparticle Concentration and Oil Volume Fraction Using Olive Oil

Olive oil was selected as the initial oil phase to screen the effects of CH/GA nanoparticle concentration and oil volume fraction (ϕ) on the emulsion characteristics. The CH/GA concentration was varied from 0.5% to 2.0% *w*/*v*, and the oil volume fraction was adjusted from 0.5 to 0.7 (Table 1). Pickering emulsions were prepared at room temperature (25 ± 2 °C) by gradually adding olive oil to the CH/GA nanoparticle suspension, followed by homogenization using a rotor–stator homogenizer at 13,500 rpm for 5–7 min [23]. The final emulsion volume was adjusted to 100 mL.

#### 2.2.3. Screening of Oil Type and Volume Fraction for Pickering Emulsion Base

Following the initial screening with olive oil, four different oils were evaluated to examine the effects of oil type on emulsion stability and appearance: olive, soybean, sunflower, and coconut oils. Two CH/GA nanoparticle concentrations (1.5% and 2.0%) and corresponding oil volume fractions (ϕ = 0.6 and 0.7) were selected based on the prior screening results (Table 2). The formulations were prepared using the same homogenization method described in Section 2.2.2.

### 2.3. Preparation of Quercetin-Loaded Pickering Emulsions

QE was incorporated at concentrations of 0.1–1% (*w*/*w*) into two optimized Pickering emulsion base formulations to identify the most suitable loading level. Briefly, QE was first dispersed in the selected oil phase using ultrasonication for 30 min under light-protected conditions to ensure uniform distribution and to minimize degradation. The resulting QE-loaded oil was then gradually added to the prepared CH/GA nanoparticle suspension, followed by homogenization using a rotor–stator homogenizer at 13,500 rpm for 5–7 min at room temperature (25 ± 2 °C). The formulation details are listed in Table 3.

### 2.4. Physicochemical Characterization of Quercetin-Loaded Pickering Emulsions

#### Morphological Observation by Confocal Laser Scanning Microscopy (CLSM)

The microstructure of the Pickering emulsions was analyzed according to a previously modified procedure using a Leica Stellaris 5 confocal microscope (Leica Microsystems Inc., Heidelberg, Germany) [24]. Each sample (1 mL) was transferred to a 1.5 mL centrifuge tube for analysis. Various samples of the Pickering emulsions were dyed with Nile Red (20 µL, 1 mg/mL in isopropyl alcohol) and Nile Blue (20 µL, 1 mg/mL in isopropyl alcohol). The dyed samples (200 µL) were placed in the hole of a concave slide and covered with coverslips. The fluorescent dyes were excited at 488 nm for Nile Red or 633 nm for Nile Blue.

### 2.5. Stability Study of Quercetin-Loaded Pickering Emulsions

#### 2.5.1. Storage Conditions

The stability was evaluated under both accelerated and ambient conditions. Accelerated testing involved heating–cooling (H/C) cycles at 4 ± 2 °C and 45 ± 2 °C for 24 h each, repeated over seven cycles (14 days). Parallel samples were stored at an ambient temperature (30 °C) for 30 days. After the storage period, the formulations were evaluated for multiple parameters, including physical appearance, microstructure using light microscopy, particle size and size distribution, QE content via HPLC, and antioxidant activity using the DPPH assay.

#### 2.5.2. Physical Appearance

Macroscopic physical changes, such as phase separation, color, and consistency, were observed visually.

#### 2.5.3. Light Microscopy

The particle morphology of the QE-loaded Pickering emulsions was examined using a light microscope (CH-2, Olympus, Tokyo, Japan). A small amount of each formulation was evenly spread onto a clean glass slide, covered with a coverslip, and observed under an upright light microscope at 10× magnification. The droplet structure, size distribution, and dispersion uniformity were assessed.

#### 2.5.4. Particle Size and Size Distribution

The average particle size and polydispersity index (PDI) of the emulsions were measured using a Zetasizer Nano ZS (Malvern Instruments Ltd., Malvern, UK) based on dynamic light scattering (DLS). Prior to analysis, each formulation was diluted 10-fold with DI water to reduce the concentration and minimize multiple scattering effects. The samples were gently vortexed to achieve uniform dispersion. Measurements were performed at 25 °C, and the results were reported as the particle size and PDI value.

#### 2.5.5. Rheological Behavior and Viscosity

The viscosity and flow behavior of the formulations were evaluated using a parallel plate rheometer (MCR 102, Anton Paar, Graz, Austria). The samples were placed on the lower plate, and a 1 mm gap was maintained between the plates. The viscosity was measured over a shear rate range of 0.1 to 150 s^−1^ at 25 °C. Both forward and reverse shear cycles were applied to assess the flow behavior. The results are reported as viscosity versus shear rate.

#### 2.5.6. Quercetin Content Determination by High-Performance Liquid Chromatography

The QE content in the Pickering emulsions and their corresponding oil-only controls was quantified using high-performance liquid chromatography (HPLC; Shimadzu Prominence, Kyoto, Japan), with modifications based on the method reported by Wipada et al. [25]. Extract samples were prepared at a concentration of 1 mg/mL by dissolving them in methanol and filtered through a 0.45 µm PTFE membrane filter prior to analysis. A 10 µL aliquot of each sample was injected into a reverse-phase HPLC system equipped with a 250 × 4.6 mm, 5 μm Capcell Pak C18 column (Shiseido Co., Ltd., Tokyo, Japan) connected to a UV detector.

The mobile phase consisted of acetonitrile and 0.1% (*v*/*v*) phosphoric acid in water at a ratio of 36:64, delivered at a flow rate of 1.0 mL/min for 10 min. The detection was performed at a wavelength of 365 nm. The target compounds were quantified using external calibration curves generated from the corresponding analytical standards.

#### 2.5.7. Antioxidant Activity Using DPPH Assay

The antioxidant activities of the QE-loaded Pickering emulsions and their respective oil-based counterparts were evaluated using a modified DPPH radical scavenging assay based on the method reported by Sainakham et al. [26]. The DPPH radical scavenging activity was examined in a 96-well plate. The samples were combined with DPPH in ethanol and incubated for 30 min at 27 ± 2 °C in the dark, and the absorbance was measured at 517 nm. The percentage of free radical scavenging activity before and after the stability test was obtained using Equation (1). The percentage change in free radical scavenging activity after the stability test was expressed using Equation (2).

(1) $$\%\;Scavenging\;activity=\left[\frac{A_{control}-A_{sample}}{A_{control}}\right]\times 100$$

where $A_{control}\;$is the absorbance of the control and $A_{sample}$ is the absorbance of the sample.

(2) $$\%\;Change=\left[\frac{{SA}_{before}-{SA}_{after}}{{SA}_{before}}\right]\times 100$$

where ${SA}_{before}$ is the percentage of free radical scavenging activity before the stability test, and ${SA}_{after}$ is the percentage of free radical scavenging activity after the stability test.

### 2.6. Cytotoxicity Test

The cytotoxicity of the QE-loaded Pickering emulsions was evaluated using the MTT assay on human keratinocyte (HaCaT) cells [25]. HaCaT cells are a spontaneously immortalized human keratinocyte line that has been widely used in studies of skin biology and differentiation. Given that the final product is intended for topical application, HaCaT cells were chosen as a relevant and well-established in vitro model for evaluating skin cytocompatibility.

Cells were cultured in Dulbecco’s Modified Eagle Medium (DMEM) supplemented with 10% (*v*/*v*) fetal bovine serum (FBS) and 1% (*w*/*v*) penicillin–streptomycin. The cultures were maintained at 37 °C in a humidified 5% CO_2_ incubator.

HaCaT cells were seeded at a density of 1 × 10^4^ cells/well in 96-well plates and allowed to attach for 24 h. The cells were then treated with various concentrations of the QE-loaded Pickering emulsions for another 24 h. After treatment, the medium was removed and replaced with a medium containing MTT solution, followed by incubation for 4 h at 37 °C. The resulting formazan crystals were solubilized using dimethyl sulfoxide (DMSO). Absorbance was measured at 560 nm using a microplate reader (Synergy H1; Agilent Technologies, Santa Clara, CA, USA). Cell viability was expressed as a percentage relative to that of the untreated control cells.

### 2.7. Measurement of Intracellular Reactive Oxygen Species

The intracellular reactive oxygen species (ROS) scavenging activity of the QE-loaded Pickering emulsions was assessed using the fluorescent probe 2′,7′-dichlorofluorescein diacetate (DCFH-DA), following a modified protocol from previous studies [27]. Upon entering the cell, DCFH-DA is deacetylated to DCFH, which is oxidized by ROS to form highly fluorescent 2′,7′-dichlorofluorescein (DCF), allowing the quantification of intracellular oxidative stress. HaCaT cells were seeded in 96-well plates at a density of 5 × 10^5^ cells/mL and incubated for 24 h at 37 °C in a humidified 5% CO_2_ incubator. The cells were then pretreated with QE-loaded Pickering emulsions for 24 h, followed by exposure to hydrogen peroxide (H2O2) at a concentration of 500 µM in serum-free DMEM for 24 h to induce oxidative stress. After treatment, the cells were washed with phosphate-buffered saline (PBS) and incubated with DCFH-DA (10 µM) for 30 min at 37 °C in the dark. The cells were then harvested, and intracellular ROS levels were quantified by measuring the fluorescence intensity using a flow cytometer (BD Accuri^TM^ C6 Plus, BD Biosciences, San Jose, CA, USA). The results were expressed as relative ROS levels (%) compared to those of the untreated control group.

### 2.8. Statistic Statistical Analysis

All experiments were performed in triplicate. Data for particle size and size distribution, QE content, percentage of free radical scavenging activity, and cell viability are expressed as mean ± standard deviation (SD). Statistical comparisons between the two groups were conducted using the paired Student’s *t*-test, with *p* < 0.05 considered statistically significant. All analyses were performed using IBM SPSS Statistics for Windows version 27.0.1 (IBM Corp., Armonk, NY, USA).

## 3. Results and Discussion

### 3.1. Effect of CH/GA Nanoparticle Concentration and Oil Volume Fraction on the Physical Appearance of Pickering Emulsions

**Figure 2:**
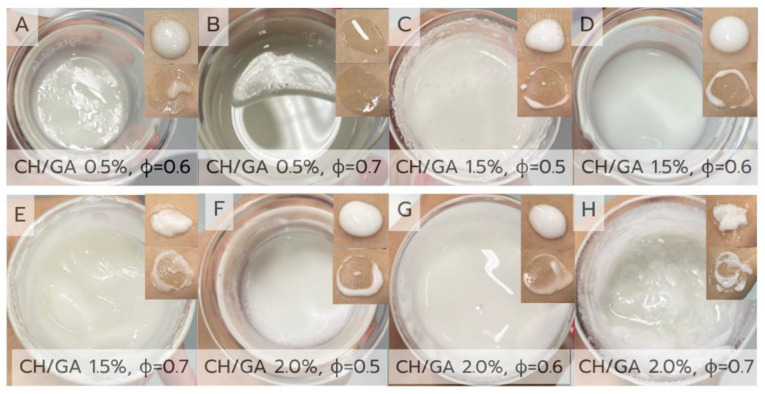
Visual appearance and spreadability of olive oil-based Pickering emulsion base formulations (**A**–**H**) prepared with varying chitosan/gum arabic nanoparticle concentrations and oil volume fractions (ϕ). Each image shows the formulation in a glass container alongside its corresponding texture when applied to the skin.

The formation and stability of Pickering emulsions depend strongly on the concentration of the stabilizing particles and the oil phase volume fraction (ϕ). In this study, CH/GA nanoparticles were employed as solid stabilizers for olive oil-in-water emulsions, with CH/GA concentrations ranging from 0.5% to 2.0% (*w*/*v*) and oil volume fractions (ϕ) of 0.5–0.7. The physical characteristics of the resulting formulations are presented in Figure 2.

At the lowest particle concentration (0.5% *w*/*v*), the emulsions (Formulations A and B) appeared fluid and unstable. Specifically, Formulation B (ϕ = 0.7) underwent immediate phase separation, as shown in Figure 2B. This distinct behavior is likely due to the significantly increased oil volume, which resulted in a larger interfacial area that could not be sufficiently stabilized by the limited number of CH/GA nanoparticles. Such instability at high oil volume fractions and low nanoparticle content is consistent with previous reports, which demonstrated that inadequate stabilizer concentrations at elevated oil fractions lead to emulsion breakdown. Moreover, as the oil volume fraction increased, the average droplet size also increased, which is an expected trend attributed to the reduced availability of nanoparticles per unit interface, limiting their ability to fully cover and stabilize smaller droplets [28]. In contrast, Formulation A (ϕ = 0.6) exhibited moderate surface oiling while maintaining partial homogeneity. Increasing the CH/GA concentration to 1.5% *w*/*v* (Formulations C–E) markedly improved both viscosity and emulsion stability. Formulations D (ϕ = 0.6) and E (ϕ = 0.7) produced uniform, cream-like emulsions with no visible signs of separation, suggesting an optimal particle loading for effective interfacial stabilisation.

Conversely, further increasing the CH/GA concentration to 2.0% *w*/*v* (Formulations F–H) led to the formation of overly thick, heterogeneous emulsions. In particular, Formulation H (ϕ = 0.7) exhibited renewed phase separation, potentially due to particle aggregation or the formation of an overly dense network within the continuous phase. These observations support the well-established role of solid particle concentration in regulating droplet size and emulsion stability [29,30] and emphasize the importance of optimizing nanoparticle loading to achieve balanced interfacial coverage and manageable rheological behavior.

In parallel, the oil volume fraction significantly influences the emulsion behavior. Formulations with moderate oil content (ϕ = 0.5–0.6) generally demonstrate superior physical stability, whereas higher oil loading (ϕ = 0.7) appears to increase internal phase pressure, leading to greater droplet collision and coalescence, particularly when the available nanoparticle coverage is insufficient to maintain interfacial integrity [31]. This suggests that emulsion stability is not determined by either nanoparticle concentration or oil content alone, but by their optimal combination.

Among all the formulations evaluated, formulation D (1.5% CH/GA, ϕ = 0.6) exhibited the most favorable physical characteristics, displaying a uniform and smooth cream texture with no evidence of phase separation. These observations align with previous reports on biopolymer-stabilized Pickering emulsions—such as those utilizing xanthan gum, cress seed gum, cellulose, or chitosan—which highlight the essential roles of adequate particle availability, favorable wettability, and strong interfacial adsorption in promoting emulsion stability [13,32,33,34].

In conclusion, a formulation incorporating 1.5% (*w*/*v*) CH/GA nanoparticles with an oil volume fraction between 0.6 and 0.7 was proposed as the optimal base for subsequent QE loading. This composition provides robust physical stability and desirable textural properties, supporting its suitability for further development as a topical delivery system.

### 3.2. Effect of Oil Type and Volume Fraction on the Physical Appearance of Pickering Emulsions

**Figure 3:**
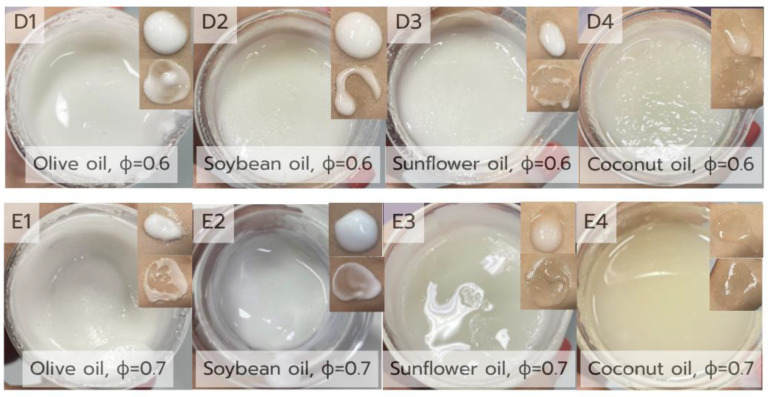
Visual comparison of Pickering emulsion base formulations prepared with different oil types and chitosan/gum arabic nanoparticle concentrations. Formulations (**D1**–**D4**) were prepared with 1.5% CH/GA and an oil volume fraction (ϕ) of 0.6, while (**E1**–**E4**) were prepared with 1.5% CH/GA and ϕ = 0.7. The oil phases used include olive oil (**D1**,**E1**), soybean oil (**D2**,**E2**), sunflower oil (**D3**,**E3**), and coconut oil (**D4**,**E4**). Each panel shows the emulsion in a glass container and its corresponding spreadability on the skin. The images illustrate differences in texture, opacity, and phase separation behavior depending on oil type and formulation parameters.

The physical stability and appearance of the Pickering emulsions were strongly influenced by the type of oil used, as illustrated in Figure 3. Emulsions were prepared using a fixed CH/GA nanoparticle concentration (1.5% *w*/*v*) with two oil volume fractions (ϕ = 0.6 and 0.7) across four different oil types: olive, soybean, sunflower, and coconut. Formulations D1–D4 (ϕ = 0.6) and E1–E4 (ϕ = 0.7) were visually evaluated as a screening step prior to QE incorporation.

At ϕ = 0.6, olive oil-based formulation D1 appeared as a smooth, homogenous cream with no evidence of phase separation, while D2 (soybean oil) displayed similar viscosity and opacity with slight surface oiling. Conversely, the sunflower (D3) and coconut oil (D4) formulations exhibited signs of instability; D3 was fluid and oily, while D4 separated rapidly, indicating insufficient interfacial stabilization. At ϕ = 0.7, the soybean oil-based formulation E2 retained moderate viscosity and visual stability, whereas the other oils (E1, E3, and E4) showed signs of thickening, oil pooling, or phase separation.

These findings highlight D1 (olive oil, ϕ = 0.6) and E2 (soybean oil, ϕ = 0.7) as the most physically stable formulations, suggesting favorable compatibility between these oil phases and the CH/GA nanoparticles. Their oil viscosity and polarity may have promoted effective nanoparticle adsorption at the oil–water interface, enabling better droplet coverage and structural integrity [35]. Moderate oil viscosity, as observed in olive and soybean oils, appears to support better emulsion formation by allowing sufficient droplet mobility for stabilizer particles to adsorb effectively at the interface. In contrast, oils with excessively low or high viscosities may hinder interfacial film formation by promoting rapid droplet movement and coalescence (as with sunflower oil) or restricting droplet deformation and particle adsorption (as with coconut oil). Furthermore, the polarity of the oil phase also plays a role in the stabilizer behavior. Although hydrophobic particles typically favor adsorption in non-polar media, studies have shown that polar oils like olive and soybean may support more effective Pickering stabilization when paired with amphiphilic biopolymer nanoparticles [36]. This is likely due to the favorable contact angles and interfacial energetics that allow the CH/GA nanoparticles to form dense, protective layers around the droplets. Thus, the superior performance of the olive oil (D1) and soybean oil (E2) systems may be attributed to their balanced viscosity and polarity profiles, which promote stable oil–water interfaces and effective nanoparticle anchoring.

Following the screening of oil types and base stability, QE was incorporated into D1 (olive oil-based) and E2 (soybean oil-based) emulsions at 0.1%, 0.5%, and 1.0% (*w*/*w*) to assess its impact on the physical properties. At 0.1% QE, D1-QE exhibited a smooth, cream-like texture and better physical homogeneity than E2-QE. However, higher QE levels (0.5% and 1.0%) resulted in overly thick, pudding-like textures in both systems, indicating reduced spreadability and flowability.

While higher QE concentrations may enhance active compound delivery, they can compromise the rheological properties and sensory attributes. Based on visual appearance, texture, and stability, a 0.1% QE concentration offers an optimal balance between formulation performance and user acceptability.

### 3.3. Morphological Observation by CLSM

**Figure 4:**
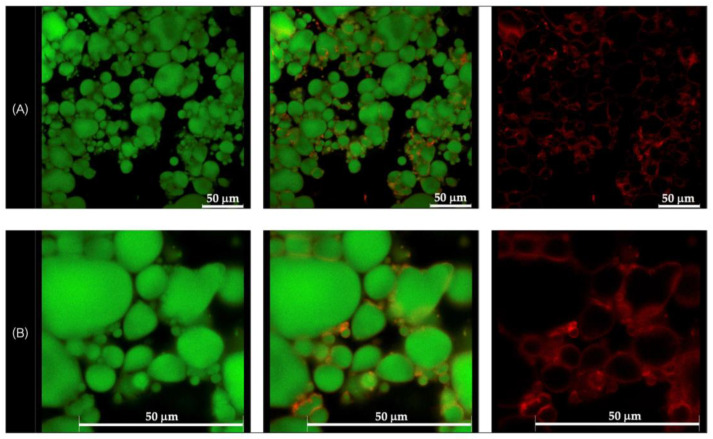
Confocal laser scanning microscopic images of the quercetin-loaded chitosan/gum arabic Pickering emulsion prepared with olive oil (D1-QE). (**A**) at magnification of 630× (**B**) at magnification of 1575× with a 50 µm scale bar. The oil droplets appear green from Nile red (on the left), whereas the adsorbed nanoparticles appear red from Nile blue (on the right). The images in the middle are an overlay of these two images (right and left).

**Figure 5:**
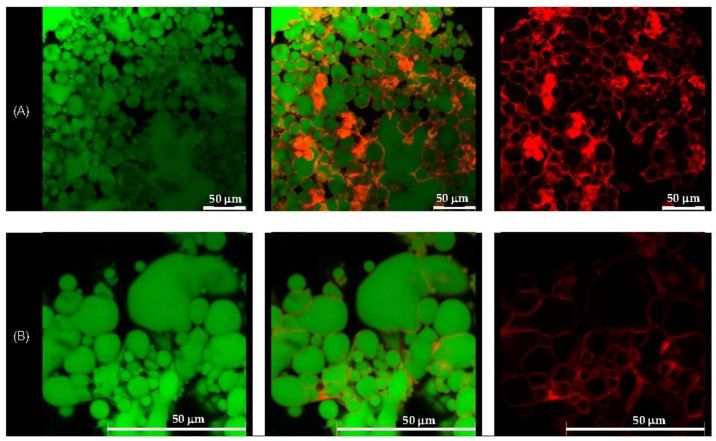
Confocal laser scanning microscopic images of the quercetin-loaded chitosan/gum arabic Pickering emulsion prepared with soybean oil (E2-QE). (**A**) at magnification of 630× with a 50 µm scale bar, (**B**) at magnification of 1575×. The oil droplets appear green from Nile red (on the left), whereas the adsorbed nanoparticles appear red from Nile blue (on the right). The images in the middle are an overlay of these two images (right and left), clearly indicating nanoparticle adsorption at the droplet interface. These results confirm the formation of a continuous interfacial layer, supporting the role of CH/GA nanoparticles in stabilizing the emulsion and preventing droplet coalescence.

Morphological analysis of the selected formulations, D1-QE (olive oil-based) and E2-QE (soybean oil-based), was performed using confocal laser scanning microscopy (CLSM) to visualize the droplet structure and interfacial particle distribution. As shown in Figure 4 and Figure 5, oil droplets were stained with Nile red (green fluorescence), and CH/GA nanoparticles were stained with Nile blue (red fluorescence). Both formulations displayed spherical oil droplets with a distinct red fluorescent rim, confirming the presence of CH/GA nanoparticles adsorbed at the oil–water interface.

The formation of a well-defined, dense interfacial layer suggests that the CH/GA nanoparticles formed a robust physical barrier around the droplets, effectively preventing coalescence and enhancing emulsion stability. This observation is consistent with the classical Pickering emulsion theory, where irreversible particle adsorption at the interface contributes to long-term droplet stabilization [37]. A similar interfacial morphology has been reported in systems stabilized with other biopolymeric nanoparticles, such as zein/gum arabic complexes, which also demonstrated resistance to phase inversion, even at elevated oil volume fractions [38].

Overall, CLSM imaging confirmed the successful localization of CH/GA nanoparticles at the oil–water interface and supported their role as effective stabilizers in both olive and soybean oil-based emulsions.

### 3.4. Stability Study

To assess the robustness of the selected formulations, both olive oil-based (D1-QE) and soybean oil-based (E2-QE) emulsions, initially identified as stable, were subjected to accelerated and ambient storage conditions. Key stability parameters were evaluated, including physical appearance, droplet morphology, particle size and distribution, rheological behavior, QE content retention, and antioxidant activity.

#### 3.4.1. Physical Appearance and Particle Morphology

**Figure 6:**
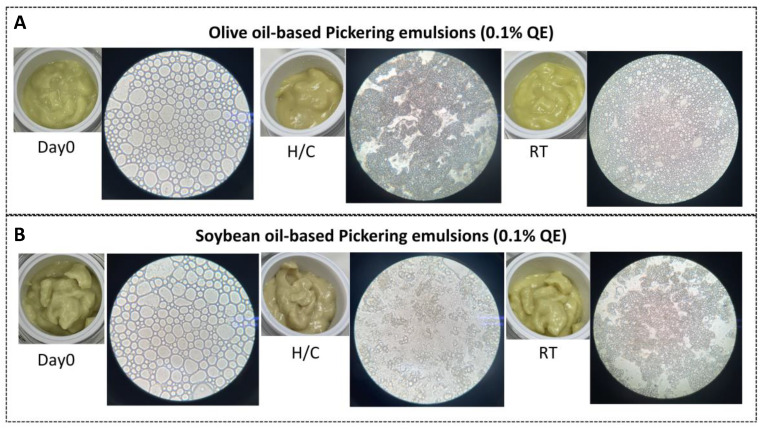
Visual and microscopic evaluation of quercetin-loaded Pickering emulsions (0.1% *w*/*w* QE) prepared with olive oil (**A**) and soybean oil (**B**) as dispersed oil phases. Each row shows the cream texture (left) alongside droplet morphology under light microscopy at 10× magnification. Emulsions were assessed at Day 0, after accelerated heating–cooling (H/C) storage, and after 30 days at room temperature (RT).

Formulations D1-QE (olive oil-based; Figure 6A) and E2-QE (soybean oil-based; Figure 6B), which demonstrated good initial stability, were subjected to accelerated and ambient storage to evaluate their physical integrity. Both emulsions initially exhibited smooth, cream-like textures and light yellow coloring, with no signs of phase separation. After H/C storage, both retained physical stability; however, a visible deepening of the yellow coloration was observed, more noticeably in E2-QE. Under ambient storage for 30 days, both formulations maintained their original appearance and color, indicating acceptable physical stability. These results suggest that while both emulsions show some sensitivity to thermal stress, they remain stable under typical storage conditions, supporting their suitability for cosmetic applications.

Microscopic examination of droplet morphology at 10× magnification (Figure 6) revealed predominantly spherical droplets with broad size distributions on Day 0 in both formulations. After H/C treatment, D1-QE maintained its spherical morphology, although with slightly smaller and more closely packed droplets, indicative of moderate droplet shrinkage or overlap. In contrast, E2-QE exhibited notable morphological disruption, with many droplets becoming irregular in shape and size, suggesting structural breakdown and weaker interfacial resilience during thermal cycling.

After ambient storage, both formulations retained a spherical morphology with moderately reduced droplet sizes, consistent with minor coalescence and gradual water loss. These findings are consistent with those of prior studies, indicating that emulsions stabilized by biopolymer-based particles can maintain their morphology under mild conditions but are vulnerable to thermal stress if interfacial anchoring is insufficient [33,34].

Overall, particle morphology analysis suggested that the olive oil-based emulsion (D1-QE) demonstrated superior structural resilience and retained interfacial integrity more effectively than its soybean oil counterpart (E2-QE). This implies that the D1-QE system may offer better performance as a long-term delivery vehicle for quercetin in topical formulation.

The superior stability observed in D1-QE may be attributed to the stabilizing behavior of the CH/GA nanoparticles, as inferred from previous studies. The proposed mechanism involves both the particle charge and wettability. Under mildly acidic conditions, chitosan carries a positive charge via protonated amine groups, while gum arabic contributes negatively charged carboxyl groups. Their interaction is known to form electrostatically stabilized polyelectrolyte complexes that create interfacial repulsion and reduce droplet coalescence. [20,21,32].

Wettability also plays a crucial role in the Pickering stabilization. Effective emulsification requires partial wettability, typically with a contact angle near 90°—to allow particles to irreversibly adsorb at the oil–water interface [34]. Based on the literature, the amphiphilic nature of CH/GA nanoparticles likely meets this requirement, enabling the formation of a robust interfacial layer. This is consistent with the findings of Sharkawy et al., who reported that CH/GA nanoparticles with optimized chitosan deacetylation could enhance interfacial stabilization and improve the delivery of hydrophobic compounds such as cannabidiol [15].

However, it should be noted that the interfacial adsorption behavior of CH/GA nanoparticles was not directly measured in this study. Future work is warranted to experimentally confirm these mechanisms through interfacial tension, contact angle, and sorption studies.

#### 3.4.2. Particle Size and Size Distribution Analysis

**Figure 7:**
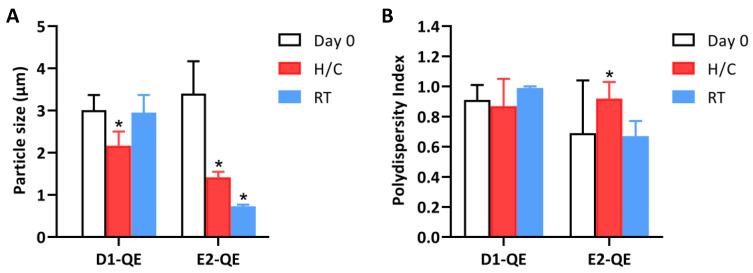
Changes in (**A**) particle size and (**B**) polydispersity index (PDI) of quercetin-loaded Pickering emulsions prepared with olive oil (D1-QE) and soybean oil bases (E2-QE) under different storage conditions. Measurements were taken at Day 0, after accelerated stability testing (H/C: heating–cooling cycles), and after 30 days of storage at room temperature (RT). * indicate statistically significant differences compared to Day 0 (*p* < 0.05).

The particle size and polydispersity index (PDI) of the QE-loaded Pickering emulsions were measured for the D1-QE and E2-QE formulations under both accelerated and ambient storage conditions (Figure 7).

On Day 0, both formulations exhibited micron-sized droplets typical of Pickering emulsions. D1-0.1 presented an average droplet size of 3.01 ± 0.36 µm with a PDI of 0.91 ± 0.10, while E2-0.1 showed a slightly larger mean size of 3.40 ± 0.77 µm and a lower initial PDI of 0.69 ± 0.35. Upon exposure to H/C cycles, both formulations exhibited a notable reduction in droplet size: D1-QE decreased to 2.17 ± 0.33 µm, and E2-QE decreased to 1.42 ± 0.13 µm. Under ambient storage, D1-QE largely retained its initial size (2.95 ± 0.42 µm), while E2-QE showed a substantial decrease to 0.73 ± 0.04 µm.

The more consistent PDI values in D1-QE across storage conditions suggest that the olive oil-based formulation better maintained droplet uniformity, while E2-QE exhibited greater fluctuation—PDI increased to 0.92 ± 0.11 after accelerated storage and decreased to 0.67 ± 0.10 under ambient conditions. These changes may reflect interfacial rearrangement or structural deformation due to environmental stress, particularly water loss or interfacial compression, rather than an improved emulsion stability.

These observations align with the findings of Saikia et al. [39], who reported that sub-10 µm organoclay-stabilized emulsions retained structural integrity under elevated thermal conditions. In general, smaller stabilizing particles promote the formation of smaller droplets and improve emulsion stability through enhanced surface area and interfacial film formation [40]. However, Ge et al. [41] emphasized that the optimal particle size (100–220 nm) is critical, as particles outside this range may not effectively anchor at the interface, thereby reducing the stabilization efficiency.

In the present study, the E2-QE formulations exhibited smaller droplets following storage, which may be favorable for topical delivery and spreadability. However, the marked size reduction could also indicate water loss or structural collapse, rather than improved stabilization. Conversely, D1-QE retained its original particle size and distribution under all storage conditions, suggesting better interfacial resilience and greater physical robustness.

These results support the selection of D1-QE as a more reliable formulation for QE delivery in cosmetic applications, particularly where long-term physical stability and environmental resilience are critical considerations.

#### 3.4.3. Rheological Behavior and Viscosity Analysis

**Figure 8:**
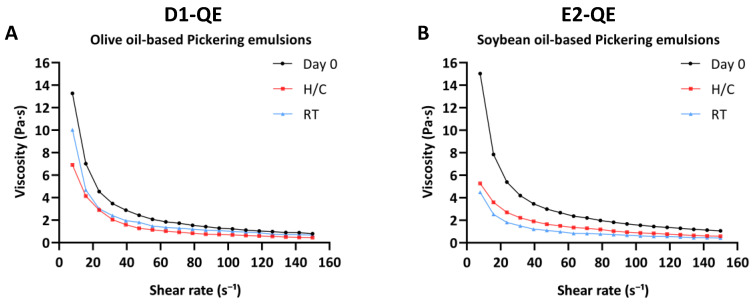
Viscosity profiles of quercetin-loaded Pickering emulsions prepared with (**A**) olive oil (D1-QE) and (**B**) soybean oil (E2-QE), measured at Day 0, after accelerated storage (H/C: heating−cooling cycles), and after 30 days at room temperature (RT).

The flow properties of topical emulsions are largely defined by their viscosity, which governs their spreadability, structural integrity, and long-term stability. Figure 8A,B present the rheological profiles of D1-QE (olive oil-based) and E2-QE (soybean oil-based) Pickering emulsions on Day 0 and after storage under accelerated and ambient conditions, respectively.

Both formulations exhibited shear-thinning behavior—a decrease in viscosity with increasing shear rate—characteristic of pseudoplastic materials, which is desirable for topical application as it enhances spreadability under applied stress while maintaining structural integrity at rest [42]. Notably, the more pronounced shear-thinning response suggests stronger internal interactions within the emulsion matrix, contributing to improved structural cohesion and overall stability [31,43].

On Day 0, D1-QE and E2-QE exhibited high initial viscosities (13.27 ± 1.52 Pa·s and 15.03 ± 1.14 Pa·s, respectively), indicating the formation of strong internal networks likely stabilized by CH/GA nanoparticles at the oil–water interface. These robust structures are essential for achieving a semisolid, cream-like consistency suitable for dermal application.

Following the H/C cycles, a marked reduction in viscosity was observed in both emulsions. D1-QE decreased to 6.92 ± 0.38 Pa·s, while E2-QE experienced a greater decrease to 5.27 ± 0.48 Pa·s. This decline likely reflects the structural rearrangement or breakdown of the interfacial network under thermal stress, with E2-QE appearing more susceptible to destabilization. Under RT, a more moderate decrease in viscosity occurred: D1-QE retained 10.03 ± 0.28 Pa·s, whereas E2-QE declined further to 4.48 ± 0.24 Pa·s, reinforcing the conclusion that E2-QE is more vulnerable to time-dependent physical degradation.

Taken together, the rheological data suggest that although the E2-QE formulation initially exhibited higher viscosity, the D1-QE formulation demonstrated superior structural resilience. Its consistently higher apparent viscosity, even after accelerated and ambient storage, correlates with the absence of phase separation, highlighting stronger internal network stability. These findings support D1-QE as a more robust and reliable candidate for topical applications, where long-term physical stability and consistent rheological performance are critical.

#### 3.4.4. Quercetin Content Determination by HPLC

**Figure 9:**
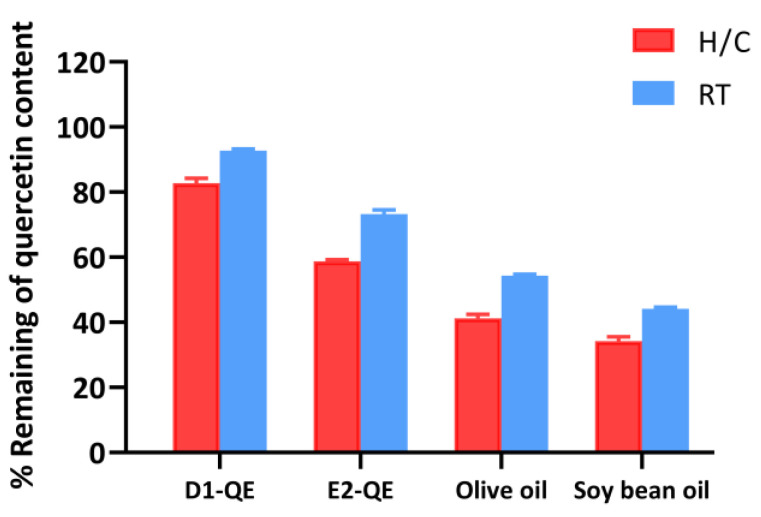
HPLC analysis of quercetin retention in emulsified and non-emulsified systems following storage under heating–cooling (H/C) and ambient room temperature (RT) conditions. Formulations include quercetin-loaded Pickering emulsions (D1-QE: olive oil-based; E2-QE: soybean oil-based) and corresponding oil controls containing quercetin without stabilizing particles (olive oil and soybean oil).

The stability of QE in various formulations was evaluated after 30 days of storage under both H/C and ambient RT conditions. As shown in Figure 9, the QE-loaded Pickering emulsions demonstrated significantly higher QE retention than their respective non-emulsified oil controls.

Among all the samples, the D1-QE formulation (olive oil-based Pickering emulsion) exhibited the highest QE retention, with approximately 82.8 ± 1.5% remaining after H/C and 92.8 ± 0.5% remaining under RT storage. In comparison, E2-QE (soybean oil-based emulsion) retained 58.8 ± 0.6% and 73.3 ± 1.3% of QE under H/C and RT conditions, respectively. Non-emulsified oils showed substantially lower retention, with the olive oil control retaining only 41.2 ± 1.2% under H/C and 54.3 ± 1.3% under RT, and the soybean oil control exhibiting the poorest stability. To ensure the correct identification of QE content in this study, the retention time was determined to be approximately 6.4 min under the specified HPLC conditions. The corresponding chromatograms are provided in Supplementary Figures S1–S4, supporting the reliability of the QE quantification results.

Notably, the sharp decline in the QE content under H/C conditions—especially in the control oils, highlights the sensitivity of QE to thermal stress. In contrast, the emulsified systems, particularly D1-QE, displayed markedly enhanced thermal stability, supporting their application in formulations requiring a prolonged shelf life and environmental resilience. Compared to the study by Wang et al. [11], where cellulose nanocrystal-stabilized Pickering gel emulsions retained 94.6% of quercetin after 14 days at 4 °C, our system retained slightly less QE. However, our evaluation was conducted under more rigorous conditions: 30 days of storage under both ambient and fluctuating thermal stress. This comparison highlights the robustness of the CH/GA-stabilized system and its relevance to dermal and cosmetic applications. The superior QE retention observed in the D1-QE formulation can be attributed to a combination of formulation-specific and interfacial factors. Olive oil, used as the oil phase in D1-QE, contains a higher concentration of intrinsic antioxidants such as tocopherols, polyphenols, squalene, sterols, and β-carotene than soybean oil [44,45]. These compounds are known to mitigate oxidative degradation and may have provided a secondary antioxidant environment that helped preserve QE during its storage. Supporting this, Ren et al. [46] reported that olive oil significantly enhances the oxidative stability of emulsions, primarily due to its high content of phenolic antioxidants, such as hydroxytyrosol and tyrosol. These compounds inhibit Ostwald ripening and improve the retention of volatile constituents within nanoemulsion systems. Although their focus was on aroma retention, the same antioxidant mechanisms apply to QE preservation in cosmetic formulations.

Furthermore, CLSM analysis revealed a denser and more uniform interfacial architecture in D1-QE, formed by the CH/GA nanoparticles. This structured interface likely limits oxygen and moisture diffusion into the dispersed phase, thereby mitigating oxidative degradation. Similar observations were reported by Sharkawy et al. [15], who demonstrated that Pickering emulsions stabilized with high-DDA CH/GA nanoparticles exhibited lower dynamic interfacial tension and brighter CLSM fluorescence, indicative of dense interfacial coverage. Han et al. [47] further confirmed that CH/GA nanoparticles enhance stability through effective interfacial adsorption and controlled release. Additionally, the polyelectrolyte complexes formed between chitosan and Arabic gum provide steric and electrostatic stabilization, preventing droplet coalescence and maintaining emulsion integrity [48].

Taken together, the enhanced QE retention observed in D1-QE can be ascribed to the synergistic effects of antioxidant-rich olive oil and dense, stabilizing nanoparticle interface. These findings highlight the functional benefits of CH/GA-stabilized Pickering emulsions and support the further development of D1-QE as a promising platform for the dermal delivery of sensitive bioactive compounds, such as quercetin.

#### 3.4.5. Free Radical Scavenging Activity

In this study, D1-QE refers to olive oil-based Pickering emulsions, and E2-QE refers to soybean oil-based Pickering emulsions. Olive oil refers to olive oil containing QE, while soybean oil refers to soybean oil containing QE. Initially, the samples exhibited a range of free radical scavenging activities, varying from 44.1% to 59.8%, as shown in Table 4. This study also examined a sample oil group that directly incorporated QE into the oil. In this research, soybean oil and olive oil were selected for the preparation of Pickering emulsion due to their antioxidant properties. The lipidic fraction of soybean oil exhibits a remarkably high radical-scavenging capacity. The key bioactive compound responsible for this activity is δ-tocopherol, which possesses strong radical-scavenging properties [49]. Due to its high content of polyunsaturated fatty acids, its oxidative stability and radical-scavenging capacity are inferior to those of certain other vegetable oils. It may be sensitive to oxidation under oxidative conditions, limiting its effectiveness in terms of long-term stability and antioxidant protection [50]. In an olive oil literature review, olive oil exhibited notable free radical scavenging activity, primarily attributed to its polyphenolic compounds, hydroxytyrosol and oleuropein. These compounds demonstrated strong antioxidant capacity against DPPH radicals, with EC_50_ values of 2.6 × 10^−7^ M and 3.6 × 10^−5^ M, respectively [51]. Virgin olive oil also exhibited significant free radical scavenging activity, as demonstrated by DPPH assays. A strong linear correlation was observed between total phenolic content and antioxidant capacity, with the DPPH method showing a high correlation coefficient R^2^ of 0.8052 [52]. These findings highlight the antioxidant potential of soybean and olive oil used in the Pickering emulsion in this study.

**Figure 10:**
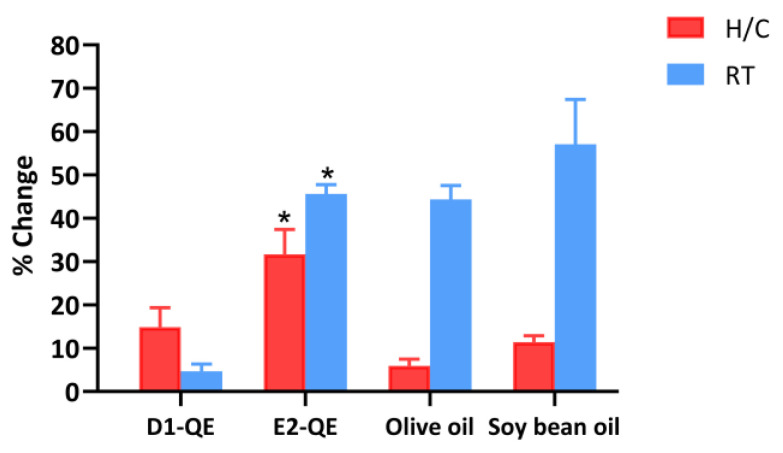
Percentage change in free radical scavenging activity of quercetin-loaded Pickering emulsions and corresponding non-emulsified vegetable oils after storage under heating–cooling (H/C) and room temperature (RT) conditions. Samples include D1-QE (olive oil-based Pickering emulsion), E2-QE (soybean oil-based Pickering emulsion), Olive oil (non-emulsified olive oil with quercetin), and Soybean oil (non-emulsified soybean oil with quercetin). Bars represent mean ± SD (*n* = 3). * indicates a statistically significant difference (*p* < 0.05) in percentage change between D1-QE and E2-QE under the same storage condition.

During the examination of the stability of D1-QE at RT, there was no significant change in the percentage of free radical scavenging activity compared to the initial value. D1-QE showed the lowest change at 4.7 ± 1.7%, compared to all other samples. Compared to the initial time, there was a significant difference at *p* < 0.05 for E2-QE, olive oil, and soybean oil. Soybean oil exhibited a significant rate of change of 57.1 ± 10.4%. The findings of this investigation indicate that D1-QE is suitable for development into a cosmetic product due to its high stability. For the H/C cycle test, there was a significant difference at *p* < 0.05 in E2-QE when compared to the initial time. E2-QE showed a significant change of 31.7 ± 5.7%. The sample that contained olive oil exhibited a considerably lesser change compared to the sample that contained soybean oil, with a significant difference at *p* < 0.05, as shown in Figure 10. The structure of the emulsion during encapsulation of QE preserves the free radical scavenging activity of the QE composition within the emulsion vesicles. The activity of QE may be influenced by structural changes in the Pickering emulsion. Pickering emulsions exhibit superior stability against oxidation compared to conventional surfactant-based emulsions, primarily due to the irreversible adsorption of solid particles at the oil–water interface. These particles form a robust interfacial layer that prevents coalescence and acts as a physical barrier to oxygen and pro-oxidant diffusion [53]. Previous studies have shown that Pickering emulsions stabilized by protein-based particles exhibit enhanced oxidative stability due to the formation of a dense interfacial layer that acts as a physical barrier against pro-oxidants. The gel-like network structure formed in high-internal-phase emulsions (HIPEs) also entraps oil droplets, shielding them from oxidative stress [54]. The use of polysaccharides, such as chitosan and pectin, improves radical scavenging at the interface. Additionally, the electrostatic charge of the interfacial layer can repel pro-oxidant ions, while increased viscosity and gel-like network structures slow down oxidative reactions. These mechanisms collectively prolong the stability of emulsified systems [55]. Consequently, olive oil may be suitable for preparing Pickering emulsions loaded with QE in this study. Pickering emulsions possess a structure that prevents the degradation of the active ingredient within the vesicle. The free radical scavenging activity of QE in these Pickering emulsions was effectively preserved by olive oil.

### 3.5. Cytotoxicity Assessment

**Figure 11:**
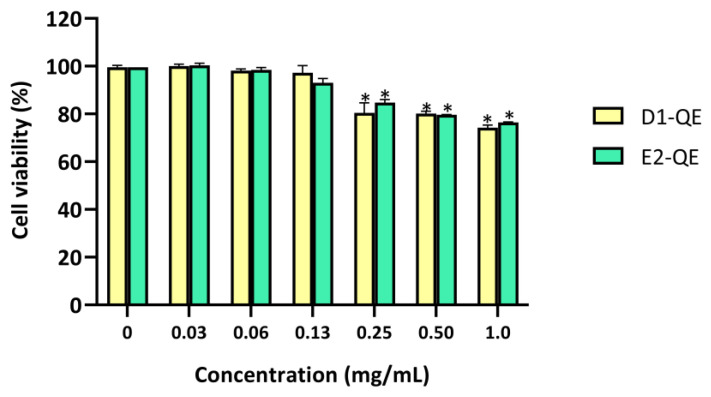
Cell viability (%) of HaCaT human keratinocyte cells after 24-h exposure to quercetin-loaded Pickering emulsions (D1-QE: olive oil-based, E2-QE: soybean oil-based) at varying concentrations (0–1.0 mg/mL), measured by MTT assay. Data are expressed as mean ± SD (*n* = 3). * indicates a statistically significant decrease in cell viability compared to the untreated control (*p* < 0.05).

The cytotoxicity of the QE-loaded Pickering emulsions was evaluated using the MTT assay on HaCaT human keratinocytes after 24 h of exposure (Figure 11). Emulsions were tested at a range of concentrations (0–1.0 mg/mL) to determine their dose-dependent effects. Both D1-QE and E2-QE exhibited high cell viability across all tested concentrations, with a clear dose-dependent trend observed. Both D1-QE and E2-QE maintained cell viability above 90% at the formulation concentration (0.13 mg/mL), with D1-QE showing slightly higher viability (97.2 ± 3.1%) than E2-QE (93.0 ± 1.9%). Notably, cell viability reached 100% at 0.06 mg/mL for both formulations, suggesting potential cell-proliferative effects at low concentrations. Although a slight decline in viability was observed at higher doses, both emulsions remained within biocompatible limits (>70%) across all concentrations. This suggests that while the emulsions are well tolerated at the formulation concentration, higher exposure may induce mild cytotoxicity. Nevertheless, both D1-QE and E2-QE remained within the accepted biocompatibility limits across all tested concentrations, supporting their potential for topical use.

### 3.6. Effect of Quercetin Loaed-Pickering Emulsions on Intracellular ROS Levels

**Figure 12:**
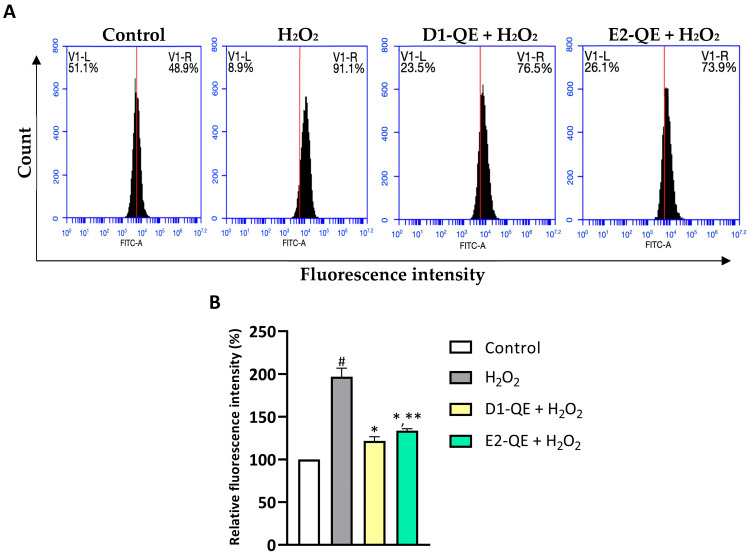
Intracellular reactive oxygen species (ROS) levels in HaCaT cells after 24-h exposure to H_2_O_2_ (500 µM) and quercetin-loaded Pickering emulsions (D1-QE and E2-QE, both at 0.1 mg/mL), assessed using H_2_DCFDA staining and flow cytometry. (**A**) Representative flow cytometry histograms showing FITC-A fluorescence intensity in control, H_2_O_2_-treated, and emulsion-treated groups. The x-axis represents fluorescence intensity (indicative of ROS levels), and the y-axis indicates the cell count. The red vertical line denotes the threshold separating low (V1-L) and high (V1-R) fluorescence intensity regions. A rightward shift (increase in V1-R%) corresponds to higher intracellular ROS levels. H_2_O_2_ treatment significantly increased ROS production (91.1% V1-R), while both D1-QE and E2-QE treatments reduced ROS accumulation in H_2_O_2_-exposed cells (76.5% and 73.9% V1-R, respectively), indicating their protective antioxidant effects. (**B**) Relative fluorescence intensity expressed as a percentage of the untreated control group. Data are presented as mean ± SD (*n* = 3). # indicates a significant increase compared to control (*p* < 0.05); * indicates a significant decrease compared to the H_2_O_2_-treated group (*p* < 0.05); ** indicates a significant difference compared to D1-QE + H_2_O_2_ group (*p* < 0.05).

Intracellular ROS levels were evaluated using the H_2_DCFDA probe and flow cytometry to assess the protective effects of QE-loaded Pickering emulsions (Figure 12). H_2_O_2_ exposure (500 µM) significantly elevated ROS levels in HaCaT cells, as indicated by a 97.02% significant increase in relative fluorescence intensity compared to the untreated control group. In contrast, pre-treatment with D1-QE and E2-QE (0.1 mg/mL) reduced intracellular ROS levels to 75.16% and 63.26%, respectively, which were significantly lower than those in the H_2_O_2_-only group. These findings demonstrate the antioxidant protective effect of QE-loaded Pickering emulsions against oxidative stress induced by H_2_O_2_. Notably, the D1-QE formulation (olive oil-based) exhibited slightly greater suppression of ROS than E2-QE (soybean oil-based), consistent with its superior QE retention and physical stability observed in previous assays. These results suggest that CH/GA-stabilized emulsions not only preserve QE’s antioxidant function but also mitigate oxidative stress at the cellular level, supporting their application in topical formulations aimed at skin protection.

## 4. Conclusions

This study successfully developed and characterized QE-loaded Pickering emulsions stabilized by 1.5% *w*/*v* CH/GA nanoparticles as solid stabilizers, demonstrating their strong potential for application in dermocosmetic formulations. Through systematic optimization of formulation parameters—including oil type, oil volume fraction, and nanoparticle concentration—the olive oil-based system (D1-QE) emerged as the most stable formulation, exhibiting favorable rheological properties and sustained antioxidant activity over time. The optimized D1-QE formulation retained more than 85% of its QE content after 30 days under both ambient and accelerated conditions. This enhanced stability is attributed to the synergistic effect of the dense interfacial architecture formed by the CH/GA nanoparticles, which restricts oxygen and moisture penetration, and the intrinsic antioxidant content of olive oil, which likely further mitigates oxidative degradation.

In addition to high retention, D1-QE preserved antioxidant efficacy and demonstrated excellent cytocompatibility in HaCaT keratinocytes, supporting its suitability for dermal applications. Collectively, these findings highlight the importance of both the oil phase and biopolymeric stabilizers in enhancing the physicochemical and biological stability of QE. The combination of biopolymeric nanoparticles and antioxidant-rich oils in Pickering emulsions offers a promising clean-label strategy for stabilizing sensitive phytochemicals, such as quercetin, in dermal formulations.

Future studies should investigate the drug release profile, ex vivo skin permeation, long-term formulation stability, in vivo safety and efficacy, and scale-up feasibility to support regulatory approval and the eventual commercialization of these clean-label biopolymer-based topical delivery systems.

## Figures and Tables

**Table 1:** Formulations of Pickering emulsion base prepared using olive oil with varying CH/GA nanoparticle concentrations and oil volume fractions.

Formulation	CH/GA Nanoparticles (% *w*/*v*)	Oil Volume Fraction (ϕ)
A	0.5	0.6
B	0.5	0.7
C	1.5	0.5
D	1.5	0.6
E	1.5	0.7
F	2.0	0.5
G	2.0	0.6
H	2.0	0.7

**Table 2:** Formulations of Pickering emulsion base prepared with different oil types and volume fractions.

Formulation	Oil Type	CH/GA Nanoparticles (% *w*/*v*)	Oil Volume Fraction (ϕ)
D1	Olive oil	1.5	0.6
D2	Soybean oil	1.5	0.6
D3	Sunflower oil	1.5	0.6
D4	Coconut oil	1.5	0.6
E1	Olive oil	1.5	0.7
E2	Soybean oil	1.5	0.7
E3	Sunflower oil	1.5	0.7
E4	Coconut oil	1.5	0.7

**Table 3:** Formulations of Quercetin-Loaded Pickering Emulsions.

Formulation	Oil Type	CH/GA Nanoparticles (% *w*/*v*)	Oil Volume Fraction (ϕ)	Quercetin Concentration (% *w*/*v*)
D1-0.1	Olive oil	1.5	0.6	0.1
D1-0.5	Olive oil	1.5	0.6	0.5
D1-1.0	Olive oil	1.5	0.6	1.0
E2-0.1	Soybean oil	1.5	0.7	0.1
E2-0.5	Soybean oil	1.5	0.7	0.5
E2-1.0	Soybean oil	1.5	0.7	1.0

**Table 4:** Percentage of free radical scavenging activity of quercetin-loaded Pickering emulsions and non-emulsified oils measured by DPPH assay at Day 0, after heating–cooling cycles, and after 30 days of room temperature storage. Formulations include olive oil-based Pickering emulsion (D1-QE), soybean oil-based Pickering emulsion (E2-QE), and their corresponding oil-only controls.

Samples	% Free Radical Scavenging		
	Day 0	Heating-Cooling	Room Temperature
D1-QE	44.1 ± 1.3	37.5 ± 1.0	42.1 ± 2.0
E2-QE	48.7 ± 5.4	33.2 ± 3.8 *	26.4 ± 2.0 *
Olive oil	59.8 ± 2.0	56.2 ± 1.1	33.3 ± 2.9 *
Soybean oil	50.2 ± 7.2	44.4 ± 6.3	21.1 ± 2.5 *

Note: Values are expressed as mean ± SD (*n* = 3). * indicates a statistically significant difference in free radical scavenging activity (*p* < 0.05) between Day 0 and post-storage conditions within the same formulation group. D1-QE = olive oil-based Pickering emulsion; E2-QE = soybean oil-based Pickering emulsion; Olive oil = olive oil containing quercetin (non-emulsified); Soybean oil = soybean oil containing quercetin (non-emulsified).

## Data Availability

The authors confirm that the data are contained within the articles.

## Supplementary Materials

The following supporting information can be downloaded at: https://www.mdpi.com/article/10.3390/polym17131871/s1, Figure S1: HPLC chromatograms showing (A) the quercetin standard at 125 µg/mL, (B) the D1-QE formulation after 30 days of storage under heating–cooling conditions, and (C) the D1-QE formulation after 30 days of storage at room temperature. Retention time for quercetin was approximately 6.4 min under the specified analytical conditions; Figure S2: HPLC chromatograms showing (A) the quercetin standard at 125 µg/mL, (B) the E2-QE formulation after 30 days of storage under heating–cooling conditions, and (C) the E2-QE formulation after 30 days of storage at room temperature. Retention time for quercetin was approximately 6.4 minutes under the specified analytical conditions; Figure S3: HPLC chromatograms showing (A) the quercetin standard at 125 µg/mL, (B) the olive oil containing QE after 30 days of storage under heating–cooling conditions, and (C) the olive oil containing QE after 30 days of storage at room temperature. Retention time for quercetin was approximately 6.4 minutes under the specified analytical conditions; Figure S4: HPLC chromatograms showing (A) the quercetin standard at 125 µg/mL, (B) the soybean oil containing QE after 30 days of storage under heating–cooling conditions, and (C) the soybean oil containing QE after 30 days of storage at room temperature. Retention time for quercetin was approximately 6.4 minutes under the specified analytical conditions.
