# Supplementary material for: Formulation and Stability of Quercetin-Loaded Pickering Emulsions Using Chitosan/Gum Arabic Nanoparticles for Topical Skincare Applications
Source: Polymers (Basel). 2025 Jul 4;17(13):1871. doi: 10.3390/polym17131871 (PMC12251611; doi:10.3390/polym17131871)
Supplement: Supplementary file 1 [file polymers-17-01871-s001.zip › polymers-3712043-supplementary.pdf]

Supplementary Information

Manuscript Title: Formulation and Stability of Quercetin-Loaded Pickering Emulsions Using Chitosan/Gum Arabic Nanoparticles for Topical Skincare Applications

Authors: Mathukorn Sainakham, Paemika Arunlakvilart, Napatwan Samran, Pattavet Vivattanaseth, and Weeraya Preedalikit*

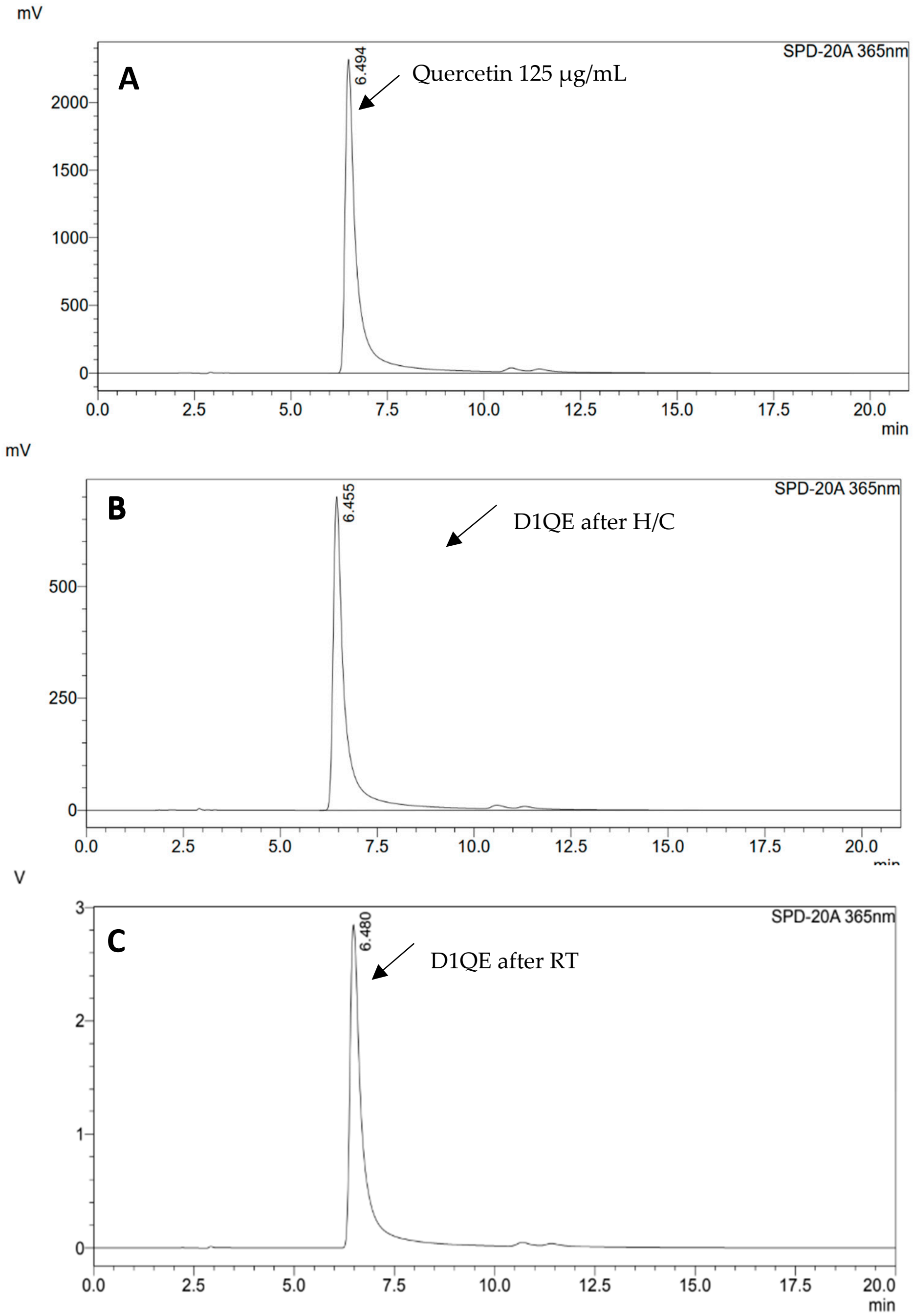

Figure S1 HPLC chromatograms showing (A) the quercetin standard at 125 μg/mL, (B) the D1-QE formulation after 30 days of storage under heating–cooling conditions, and (C) the D1-QE formulation after 30 days of storage at room temperature. Retention time for quercetin was approximately 6.4 minutes under the specified analytical conditions.

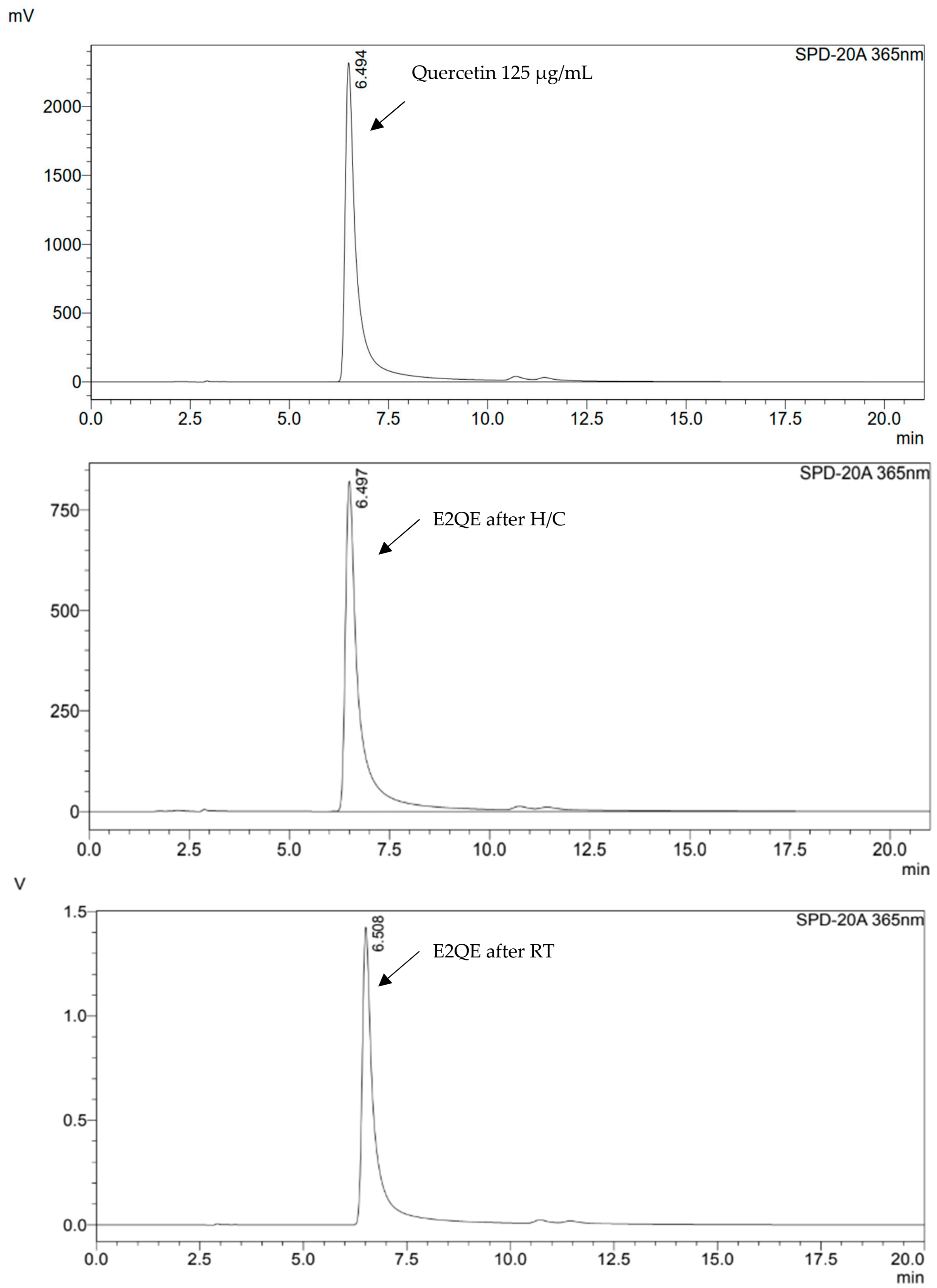

Figure S2 HPLC chromatograms showing (A) the quercetin standard at 125 µg/mL, (B) the E2-QE formulation after 30 days of storage under heating–cooling conditions, and (C) the E2-QE formulation after 30 days of storage at room temperature. Retention time for quercetin was approximately 6.4 minutes under the specified analytical conditions.

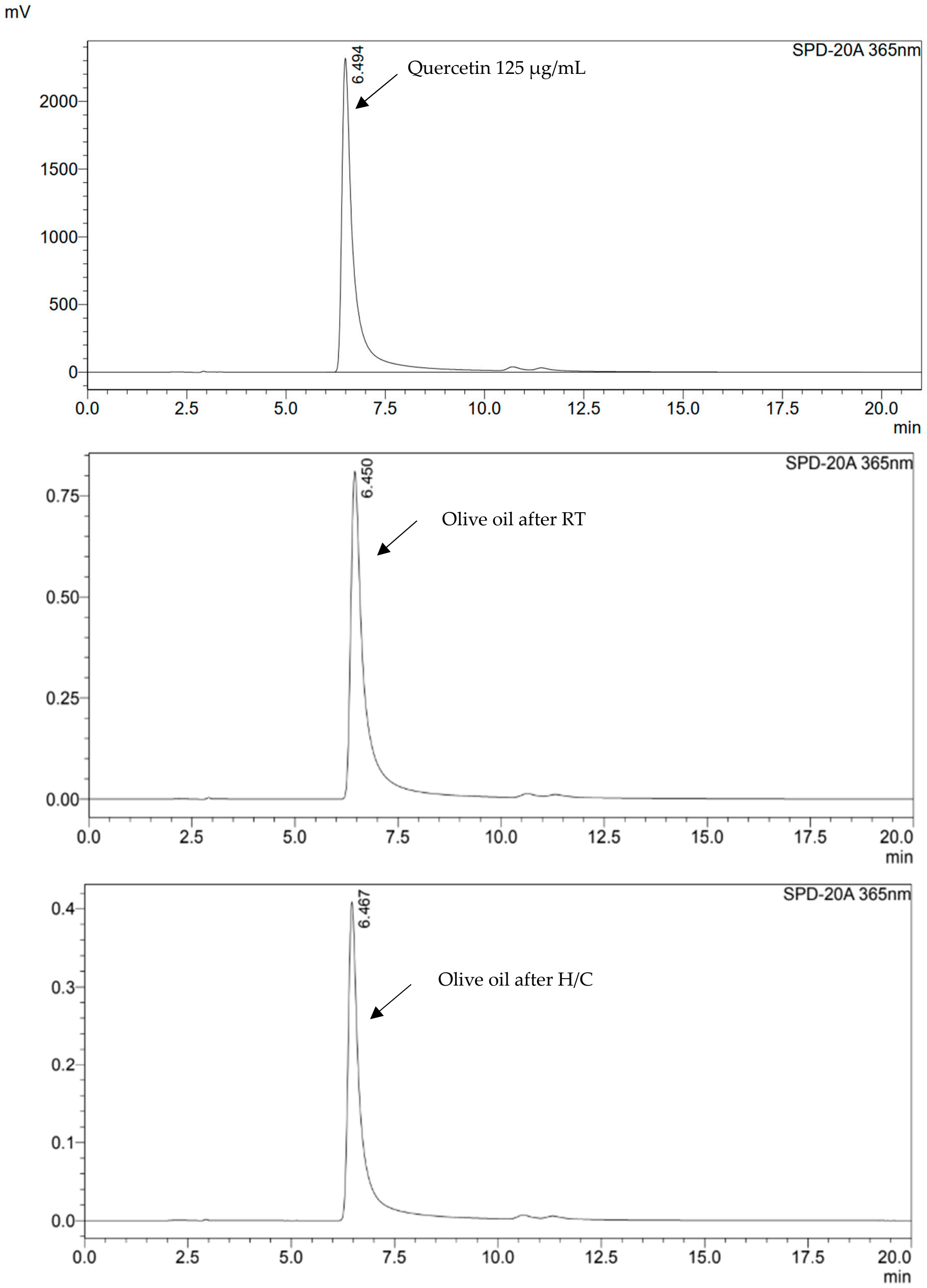

Figure S3 HPLC chromatograms showing (A) the quercetin standard at 125 µg/mL, (B) the olive oil containing QE after 30 days of storage under heating–cooling conditions, and (C) the olive oil containing QE after 30 days of storage at room temperature. Retention time for quercetin was approximately 6.4 minutes under the specified analytical conditions.

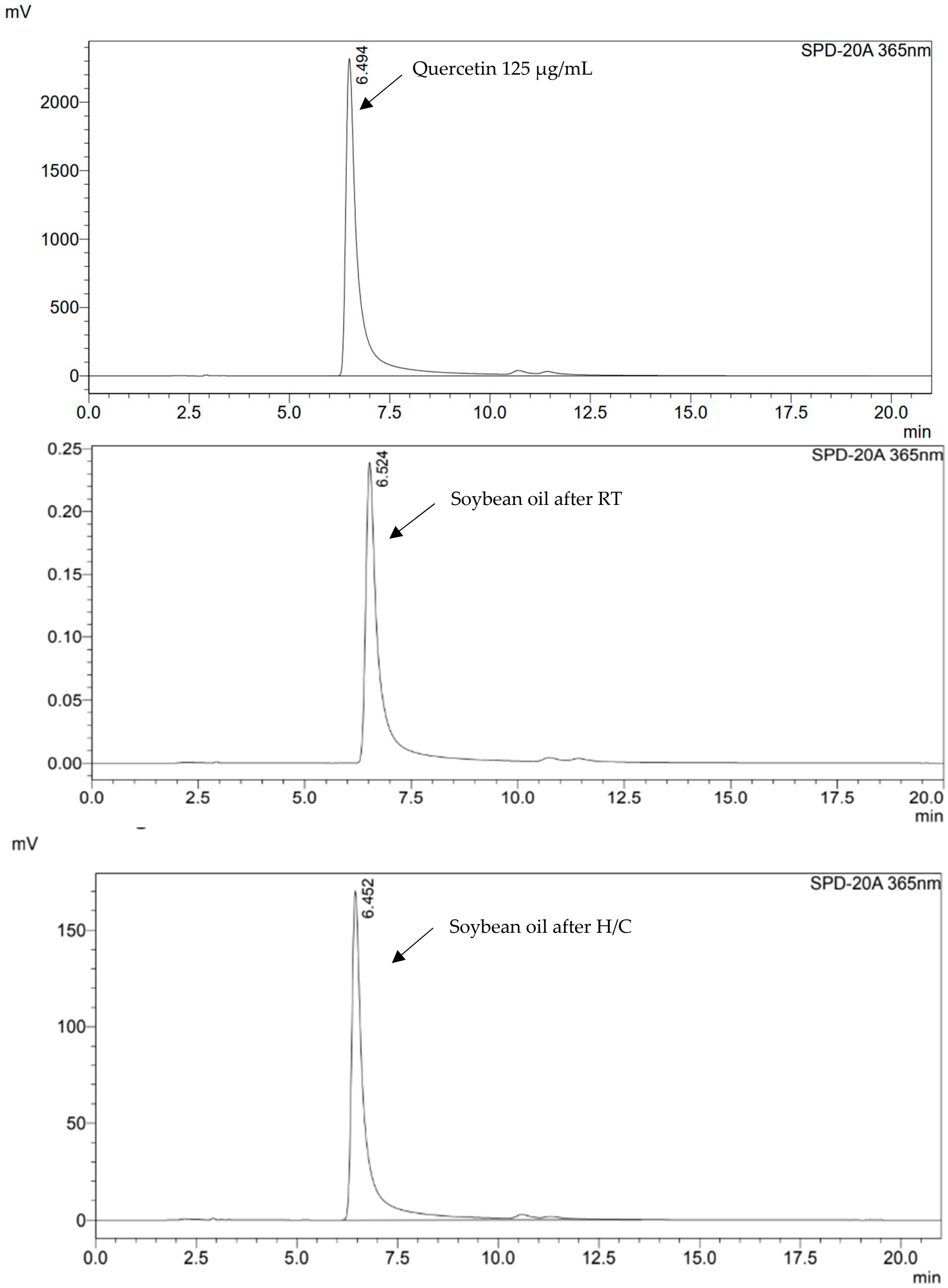

Figure S4 HPLC chromatograms showing (A) the quercetin standard at 125 µg/mL, (B) the soybean oil containing QE after 30 days of storage under heating–cooling conditions, and (C) the soybean oil containing QE after 30 days of storage at room temperature. Retention time for quercetin was approximately 6.4 minutes under the specified analytical conditions.
